# Hydrogel scaffolds in the treatment of spinal cord injury: a review

**DOI:** 10.3389/fnins.2023.1211066

**Published:** 2023-05-31

**Authors:** Manqi Cai, Liji Chen, Tao Wang, Yinru Liang, Jie Zhao, Xiaomin Zhang, Ziyi Li, Hongfu Wu

**Affiliations:** ^1^Dongguan Key Laboratory of Stem Cell and Regenerative Tissue Engineering, The First Dongguan Affiliated Hospital, Guangdong Medical University, Dongguan, China; ^2^Department of Surgery, The Third Hospital of Guangdong Medical University (Longjiang Hospital of Shunde District), Foshan, China; ^3^The Second Clinical Medical College, Guangdong Medical University, Dongguan, China

**Keywords:** hydrogel, spinal cord injury, composite hydrogel, nerve repair, tissue repair

## Abstract

Spinal cord injury (SCI) is a disease of the central nervous system often caused by accidents, and its prognosis is unsatisfactory, with long-term adverse effects on patients’ lives. The key to its treatment lies in the improvement of the microenvironment at the injury and the reconstruction of axons, and tissue repair is a promising therapeutic strategy. Hydrogel is a three-dimensional mesh structure with high water content, which has the advantages of biocompatibility, degradability, and adjustability, and can be used to fill pathological defects by injectable flowing hydrophilic material *in situ* to accurately adapt to the size and shape of the injury. Hydrogels mimic the natural extracellular matrix for cell colonization, guide axon extension, and act as a biological scaffold, which can be used as an excellent carrier to participate in the treatment of SCI. The addition of different materials to make composite hydrogel scaffolds can further enhance their performance in all aspects. In this paper, we introduce several typical composite hydrogels and review the research progress of hydrogel for SCI to provide a reference for the clinical application of hydrogel therapy for SCI.

## 1. Introduction

Spinal cord injury (SCI) is a disease of the central nervous system with serious condition and poor prognosis, which is often caused by accidental trauma such as traffic accident, fall from height, impact, etc. It may also be triggered by inflammation or tumor. SCI can lead to severe sensory, motor or autonomic dysfunction, which greatly reduces the quality of life of patients and imposes a heavy economic burden on families and society. Currently, the clinical treatment of SCI includes traditional surgical therapy and systemic administration of drugs, but the therapeutic effect is not satisfactory. Due to the limited crossover with the blood-spinal cord barrier, oral or intravenous drugs cannot produce the desired local effect (Shultz and Zhong, 2021; Sanchez-Dengra et al., 2023). Cell transplantation and *in situ* drug delivery therapies are more preferable for treatment options. Cell transplantation and molecular drug therapies can provide cellular and nutritional support to the injured area (Ma et al., 2018; Lai et al., 2019). However, due to the lack of bridging support in the injured area, transplanted cells or active factors are difficult to colonize due to cerebrospinal fluid circulation, resulting in cells and active factors cannot work effectively (Sensharma et al., 2017). Rapid release and clearance of fluid is the major problem for local drug delivery (Silva et al., 2021).

Hydrogel is a polymeric material with a three-dimensional network structure and with water as the dispersion medium, which is biocompatible and degradable (Yuan et al., 2021a). The hydrogel itself can mimic the natural extracellular matrix, filling the lesion area and improving the microenvironment of the damaged area, providing contact guidance for axonal regeneration. It can also act as a carrier of seed cells and active factors to help seed cell survival and proliferation, promote reconnection of damaged spinal cord tissue, help bridge gaps and reconstruct nerve conduction at the lesion site, and facilitate continuous drug delivery (Silva et al., 2021; Liu Y. et al., 2023). In this paper, we review the research progress of SCI repair using hydrogels for the clinical application of hydrogel therapy for SCI.

## 2. SCI and treatment

**Figure 1:**
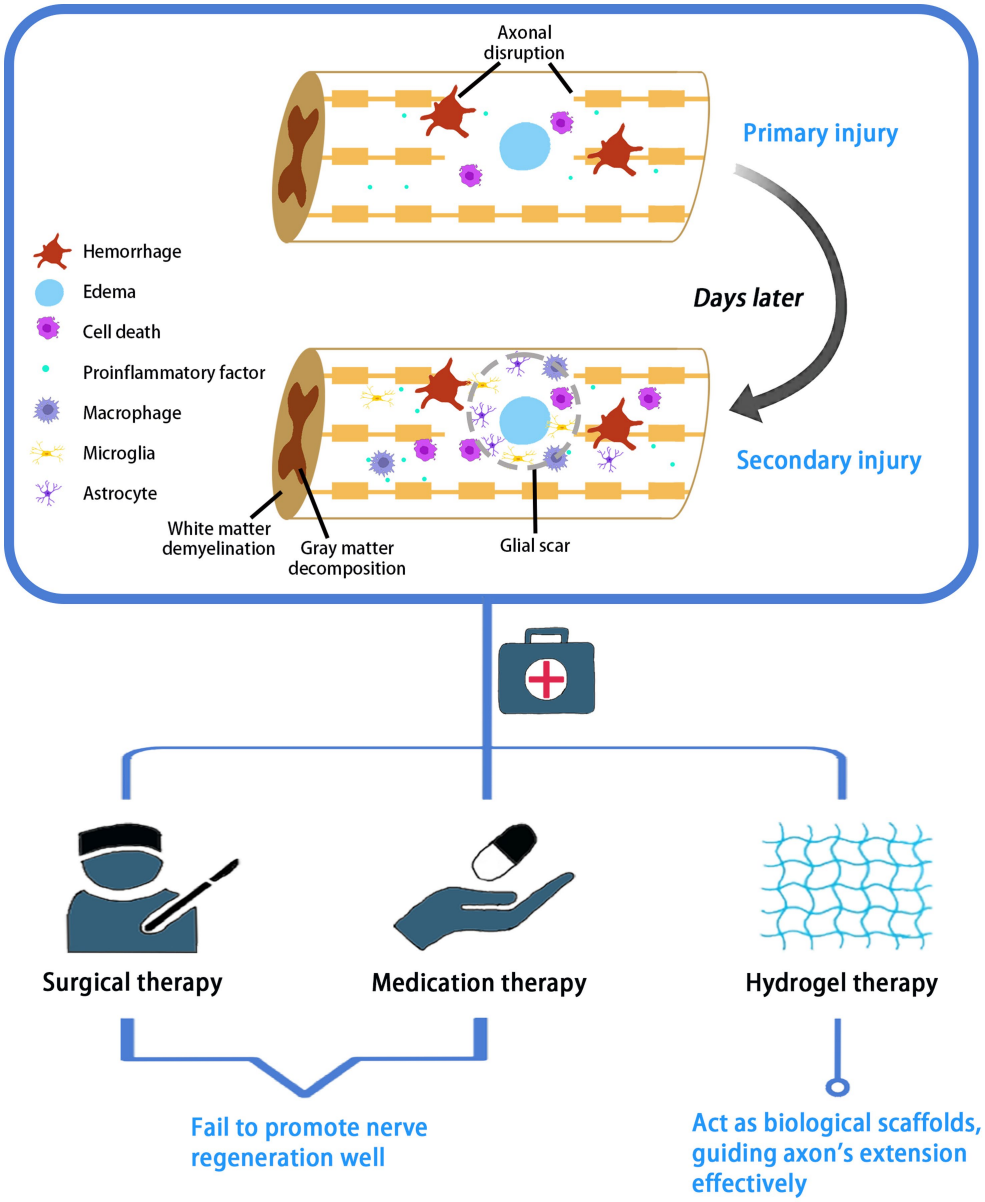
Pathological process and treatment of SCI. SCI pathologically consists of two stages: primary and secondary injury. Current treatment options for SCI include surgery, medication and hydrogel therapies.

SCI is a severe central nervous system injury often caused by external trauma (Ahuja et al., 2017). Due to neuronal death and axonal disruption after injury, patients will suffer from lifelong motor and sensory impairment and even end up dying prematurely. Traumatic SCI consists of two stages in pathology, namely primary and secondary injury. Primary injury leads to immediate mechanical rupture and dislocation of the spine, resulting in compression or transection of the spinal cord, causing damage to neurons and oligodendrocytes and disruption of the vascular system and blood-spinal cord barrier. The spinal cord lesion is further exacerbated by massive cell necrosis or apoptosis, ionic imbalance, and inflammatory response (Wang et al., 2015; Wang J. et al., 2022), which triggers persistent secondary SCI. The release of ATP, DNA and potassium ions after cell necrosis activates microglia to secrete pro-inflammatory factors, and a large number of macrophages, microglia and astrocytes are attracted to infiltrate the damaged area, further contributing to cell death, gray matter breakdown, white matter demyelination, and the formation of glial scar, a physical barrier that limits axonal growth (Anderson et al., 2016; Dias et al., 2018; Anjum et al., 2020). Secondary spinal cord injuries produce further chemical and mechanical damage to the spinal cord tissue, which is more serious than that caused by the primary injury (Ham and Leipzig, 2018; Figure 1).

Current management strategies for SCI involve early surgical decompression and fixation, the use of vasopressor medications for mean arterial blood pressure augmentation to improve spinal cord perfusion, and corticosteroids (Karsy and Hawryluk, 2019). Corticosteroids, such as methylprednisolone, are administered in high doses orally or intravenously to upregulate the release of anti-inflammatory cytokines and reduce oxidative stress. In addition, dehydrating agents are often used to treat spinal edema, such as intravenous mannitol or intravenous furosemide. However, the above-mentioned surgical and pharmacological treatments cannot fundamentally address the problem of nerve regeneration. Relative drugs have been widely used in clinic, but their efficacy in nerve injury is still unsatisfactory caused by drug toxicity and drug delivery (Kabu et al., 2015). Comparatively, stem cell transplantation and local injection of growth factors or small molecule drugs are superior treatment options. Transplanted stem cells in the area of injury can promote myelin regeneration and rebuild the interrupted neural circuits (Figure 1). This therapy holds great promise, but potential problems remain to be considered, such as immune rejection, tumor formation, and inability of the cells to sustain their action in the damaged area. Local injections also suffer from explosive drug release and rapid degradation (Hou et al., 2022).

To deal with these problems, tissue engineering studies using biomaterials as a transport system to load stem cells or drugs have been widely carried out. Factors or stem cells are loaded into the biomaterial to form a biological scaffold with specific functions, which is then implanted into the damaged area. The biological scaffold acts as a bridge to connect the two ends of the injured nerve, regulate the microenvironment of the injured area, provide support for cell growth, adjust the drug release rate. Hence, it plays an important role in the delivery of cells and drugs and promotes the regeneration of myelin sheath and recovery of nerve function (Sousa et al., 2023; Szymoniuk et al., 2023). According to the bionic principle and therapeutic requirements, the ideal tissue engineering scaffold material needs to have the following characteristics: (1) Good tissue biocompatibility: low biocompatibility can cause toxic reactions and immune inflammation in humans, and high biocompatibility can reduce graft failure caused by rejection. (2) Specific mechanical strength: the scaffold should have suitable flexibility to adapt to the movement of the body and avoid physical damage caused by malposition. (3) Appropriate degradation rate: the degradation rate of biodegradable scaffolds should be regulated based on the rate of nerve regeneration. Too fast degradation of scaffolds cannot provide sufficient support for nerve injury, while too low degradation rate will also hinder the regeneration and reconstruction of injury. (4) Three-dimensional configuration with appropriate pore size and porosity: the porosity and pore size also determine the flow of different components that promote or inhibit nerve regeneration. In addition, three-dimensional structure can mimic the role of neuronal extracellular matrix, promote cell connectivity, facilitate scaffold surface material exchange and axonal growth, and cell adhesion growth. (5) High surface area to volume ratio: it facilitates cell adhesion, growth, and cell-specific gene expression (Ashammakhi et al., 2019; Bartlett et al., 2020; Kaplan and Levenberg, 2022; Liu W. et al., 2023). The hydrogel has unique advantages due to the above-mentioned properties and can overcome the limitations of surgical therapy, medication therapy, and cell therapy, opening up new ideas for the treatment of SCI.

## 3. Application of hydrogels in SCI repair

**Figure 2:**
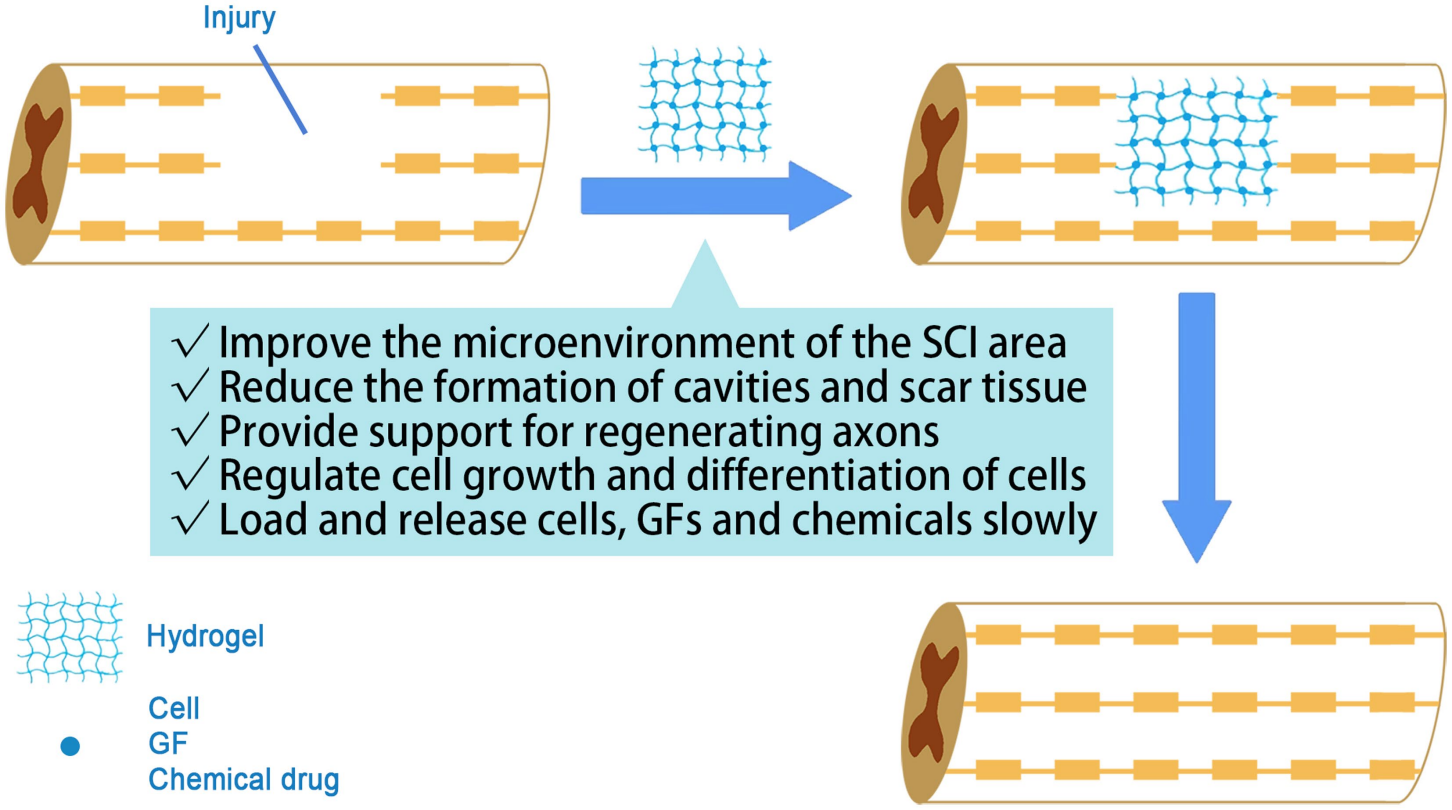
The role of hydrogel in SCI treatment. The hydrogel acts as a biological scaffold, guiding axonal extension and controlling drug release.

In the tissue engineering for the treatment of SCI, the role of hydrogels mainly includes the following points: Firstly, hydrogels provide three-dimensional spatial support for neuronal regeneration and axonal extension, which is conducive to cell growth, proliferation and migration and promotes neural reconstruction (Chedly et al., 2017; Silva et al., 2023). Secondly, the hydrogel itself act as a carrier to load stem cells to the site of injury, reducing cell loss to surrounding tissues and off-targeting (Peng et al., 2023). Thirdly, hydrogels slowly release bioactive factors or chemical drugs to achieve a sustained and stable release for better performance (Luo et al., 2021; Figure 2).

Loading factors with hydrogels and controlling their release can be achieved by simple physical co-mingling. The bioactive factors are mixed directly with the biomaterial and the factors are released gradually as the material degrades. It has been found that pore size of hydrogel and stiffness are inversely proportional. To achieve sustained release of the drug, hydrogels with sufficiently small pore size are required. Such hydrogels, however, are too stiff and can easily cause damage to the soft and fragile spinal cord tissue (Chiu et al., 2013). Another route is affinity-based drug delivery (Vulic and Shoichet, 2014; Nambiar and Schneider, 2022). The hydrogel material is made to adsorb with the factors based on properties such as charge and hydrophobicity of the substance, thus immobilizing the factors on the surface and inside of the hydrogel. Drug release from affinity-based delivery systems can be controlled by modulating the affinity between the drug and its carrier and the ratio of the drug to the binding site (Mohtaram et al., 2013). Zhao et al. prepared GDNF-loaded heparin-Poloxam hydrogels based on the binding affinity of heparin to treat animals with SCI, which showed improved histological and functional outcomes (Zhao et al., 2017). It is also possible to modify peptide or protein-based drugs to equip the hydrogel with binding affinity, but it must be ensured that the introduced chemical coupling does not affect the biological activity of the drug molecule (Shultz and Zhong, 2021).

Hydrogels used for SCI repair contain natural hydrogels and synthetic hydrogels. For the similarity and compatibility with biological tissues, the study of hydrogel materials in tissue engineering started with naturally occurring macromolecules in biological tissues, including proteins (collagen, fibronectin, fibronectin, silk proteins, etc.) as well as polysaccharides (including agarose, alginate, cellulose, xyloglucan, etc.; Catoira et al., 2019; Wu et al., 2022; Sharma et al., 2023). These natural hydrogels are biocompatible, enzymatically degradable, porous, soft and enable cells to adhere and migrate (Taghipour et al., 2020). It was reported that natural hydrogels can promote axonal regeneration by bridging the axon bundles separated from the injured area through a similar action of the peripheral nerve endoneurium and the nerve bundle membrane (Hejčl et al., 2008).

Hydrogel scaffold should provide suitable pores for cell migration and blood vessel formation. Natural hydrogels have a fixed structure and are generally difficult to adjust, whereas synthetic hydrogels are superior in regulating the size of the pore (Gomez-Florit et al., 2020; Lyu and Azevedo, 2021). Compared to hydrogels origin from natural ubstances, synthetic materials offer a wider scope for designing and controlling material properties (Horne et al., 2010). In addition, the use of artificially designed and synthesized biocompatible materials can significantly reduce the risk of allergy or human immune rejection, and synthetic materials can be easily produced on a large scale. Currently, the raw materials of synthetic hydrogel commonly used in tissue engineering include polyacrylic acid and its derivatives, polyvinyl alcohol, and polyesters (Ranganathan et al., 2018).

### 3.1. Composite hydrogel scaffolds

Natural hydrogels are defective in terms of adjustability, and simple synthetic hydrogels are poor in terms of cell adhesion, so attempts have been made to encapsulate synthetic polymers with natural polymer monomers to form natural polymer-synthetic composite hydrogels, thus improving cell adhesion and cell activity. Single-component hydrogels also have the disadvantage of poor mechanical properties, which to some extent limits their application in tissue engineering, so studies have attempted to enhance the physicochemical properties of scaffolds by adding nanoparticles and nanotubes to hydrogels to prepare composite scaffolds. Besides, simple hydrogels are still slightly inadequate in terms of sustained drug release performance, and thus some studies have tried to solve this problem by preparing slow-release microsphere composite hydrogel scaffolds.

#### 3.1.1. Natural-synthetic composite hydrogel scaffolds

Natural-synthetic composite hydrogels are interpolymer complexes in which block copolymerization or physical interaction occurs between synthetics and natural polymers. These composite hydrogels combine the biocompatibility of natural hydrogels with the mechanically tunable properties of synthetic hydrogels. For example, the addition of polylysine to poly (ethylene glycol) (PEG; Wei et al., 2021), or chitosan to methacrylamide can increase the viscosity of the material (Saleem et al., 2022).

Gelatin is widely used in tissue engineering and drug delivery systems because of its high hemocompatibility, biodegradability and no other by-products after *in vivo* degradation, non-immunogenicity, and the same components and biological properties as collagen. Wang et al. designed a hydrogel composed of gelatin-hydroxyphenylpropionic acid (Gtn-HPA) conjugates formed by partial oxidative coupling of 3,4-hydroxyphenylpropionic acid catalyzed by H_2_O_2_ and horseradish peroxidase. It was found that the proliferation rate of mesenchymal stem cells (MSCs) increased as the stiffness of the hydrogel decreased, and the neurogenesis of MSCs was also strongly affected by the stiffness. The hardness of this material can be modulated by changing the concentration of H_2_O_2_ without changing the concentration of the polymer precursor, thus controlling the proliferation rate and differentiation of MSCs in gelatin (Wang et al., 2010).

Methacrylated gelatin (GelMA), obtained by preparing methacrylic anhydride with gelatin, is a photosensitive biohydrogel material (Kurian et al., 2022). Cai et al. prepared a conductive, longitudinally grooved GelMA-MXene nerve conduit and explored its role in SCI repair. It was found that the grooved GelMA-MXene effectively promoted the adhesion, directed proliferation and differentiation of neural stem cells (NSCs) *in vitro*. Its ability to effectively repair complete transection of SCI was demonstrated when GelMA-MXene loaded with NSCs was implanted in the injured spinal cord site. This group exhibited significant neural recovery and significantly higher BBB scores compared to other groups. Therefore, GelMA-MXene with microgroove structure and loaded NSC is a promising strategy for the treatment of SCI (Cai et al., 2022).

#### 3.1.2. Nanoparticle composite hydrogel scaffolds

Hydrogels also are combined with nanoparticles (organic polymer nanoparticles, inorganic nanoparticles, and metal/metal oxide nanoparticles) to obtain more excellent properties (Wang Y. et al., 2022). Organic polymer nanoparticles include dendritic macromolecules and hyperbranched polyesters. Inorganic nanoparticles include hydroxyapatite, silica, silicate and calcium phosphate. And metal/metal oxide nanoparticles include gold, silver and iron oxide. Among them, metal/metal oxide nanoparticles are particularly favored because of their corrosion resistance, oxidation resistance, high specific surface area, surface chemistry and functionalization (Tan et al., 2019).

Studies have shown that the incorporation of magnetic Fe_3_O_4_ nanoparticles into chitosan or polyethylene glycol hydrogels can promote the survival and differentiation of bone marrow mesenchymal stem cells (Tan et al., 2019; Barrett-Catton et al., 2021). Li et al. prepared MnO_2_ nanoparticles-dotted hydrogels (MnO_2_ NPs) by dispersing MnO_2_ nanoparticles in peptide-modified hyaluronic acid hydrogels. MnO_2_ NPs alleviated the oxidative environment, which effectively improved the viability of MSCs. MSCs transplanted in multifunctional gels produced significant motor function recovery in a large span rat spinal cord transection model and induced *in vivo* integration of implanted MSCs as well as neural differentiation, resulting in efficient regeneration of central neurospinal tissues (Li L. et al., 2019).

Zinc oxide nanoparticles (ZnO NPs) have been widely reported to achieve satisfying anti-inflammatory functions. Lin et al. constructed a novel ZnO NPs-Gel composite hydrogel and detected the antioxidant markers of SOD, GSH, Nrf2 and HO-1 by ELISA and protein blotting, and demonstrated that the hydrogel effectively down-regulated ROS intensity and improved the oxidative microenvironment at the injury site. In addition, apoptosis of the damaged spinal cord was inhibited. Thus, metal oxide nanoparticle composite hydrogels represent a promising strategy for the treatment of CNS disorders by comprehensively modulating the complications of the pathological microenvironment (Lin et al., 2021).

#### 3.1.3. Nanotube-composite hydrogel scaffolds

Nanotubes are widely used in biomedical fields for their low toxicity, high biocompatibility and environmental friendliness. Due to their stable tubular morphology, unique charge distribution and crystal structure, they can be dispersed in hydrogels. Studies have shown that the combination of nanotubes and chitosan has little effect on the pore structure but increases the number of pores, which facilitates cell survival (Waris et al., 2022). Different studies combined elosite nanotubes with different hydrogels such as chitosan, alginate and collagen to form nanotube composite hydrogels. These studies confirmed the biocompatibility and safety of the composite hydrogels and showed that the addition of elosite nanotubes could improve the mechanical strength of the composite hydrogels (Kazemi-Aghdam et al., 2021; Machowska et al., 2022).

Some studies have incorporated carbon nanotubes into gelatin-methacryloyl prepolymer formulations to form biocompatible hydrogels after UV irradiation. The photocrosslinking process of the prepolymer provided delivery of the formulation at the site of injury. This composite hydrogel scaffold improved the reconnection process, provided a site for nerve cell development, and delivered methononin to improve inflammation. It was found that the incorporation of carbon nanotubes effectively promoted neurite growth (de Vasconcelos et al., 2020).

#### 3.1.4. Microsphere composite hydrogel scaffolds

Effective and accurate drug delivery to SCI tissues is crucial to restore neurological deficits. Burst release phenomenon may lead to unexpected side effects. Hydrogels perform well in supporting cell adhesion and guiding cell migration to the affected area, but there is still room for improvement in sustained drug release. Sustained-release microspheres (MS), on the other hand, facilitate sustained and stable drug release (Wang et al., 2011) and also improve the precision of drug delivery (Wu et al., 2023). Therefore, a lot of studies have combined sustained-release MS with hydrogels to form composite hydrogel scaffolds capable of sustained drug release. Wen et al. prepared HA hydrogels with a longitudinal multichannel structure and carried poly (lactic-co-glycolic acid) (PLGA) sustained-release MS encapsulated with vascular endothelial growth factor (VEGF) to form HA composite hydrogels. The HA composite hydrogel was implanted into the SCI area of rats as the experimental group, and the rat model of SCI without the implantation material and the HA hydrogel implantation alone were used as the control group. It was found that the composite hydrogel integrated well with the spinal cord tissue, induced vascular and nerve fiber regeneration, and could promote the recovery of motor function after SCI in rats (Wen et al., 2016).

Zhang et al. developed and optimized injectable PLGA MS loaded with melatonin (Mel), which was further mixed with Laponite hydrogel to form a composite hydrogel scaffold to reduce the sudden release of MS. The MS was able to achieve stable and prolonged Mel release as well as synergistic Lap hydrogel to repair neural function in SCI by *in situ* injection (Zhang et al., 2021).

Composite hydrogels with the addition of different materials possess corresponding advantages, including mechanical properties, electrical conductivity, oxidation resistance, and extended release ability (Table 1). Most of the current composite hydrogels have only one of these advantages, and there is no one that combines all of them. However, it is not a simple addition of the strengths of each, but also needs to consider the interaction of different additives and the overall impact on the composite hydrogels. Research on the preparation of composite hydrogels with better overall performance is still ongoing.

**Table 1:** Different composite hydrogels with their advantages and disadvantages.

Composite hydrogels	Type of additive	Advantages	Disadvantages
Gtn-HPA (Wang et al., 2010)	Synthetic organics	Adjust the hydrogel stiffness by changing the concentration of H_2_O_2_	Slow-release performance to be improved; no electrical conductivity; no antioxidant capacity
Grooved GelMA-MXene (Cai et al., 2022)	Synthetic organics, nano-materials	Increase electrical conductivity and promote nerve repair; direct cell migration	Slow-release performance to be improved; no antioxidant capacity
MnO_2_ NPs-HA (Li L. et al., 2019)	Nanoparticles	Mitigate the oxidative environment and improve the viability of MSCs	Slow-release performance to be improved; no electrical conductivity
ZnO NPs-Gel (Lin et al., 2021)	Nanoparticles	Reduce ROS production and improve pathological microenvironment	Slow-release performance to be improved; no electrical conductivity
GelMA-CN (De Vasconcelos et al., 2020)	Nanotubes	Conductive, promoting neuronal growth	Slow-release performance to be improved; no antioxidant capacity
HA-MS(PLGA) (Wen et al., 2016)	Microspheres	Sustained drug (VEGF) release for improved drug utilization	No oxidation resistance; no electrical conductivity
Lap-MS (PLGA) (Zhang et al., 2021)	Microspheres	Achieve stable and sustained Mel release for improved drug utilization	No oxidation resistance; no electrical conductivity

### 3.2. Smart hydrogels

**Figure 3:**
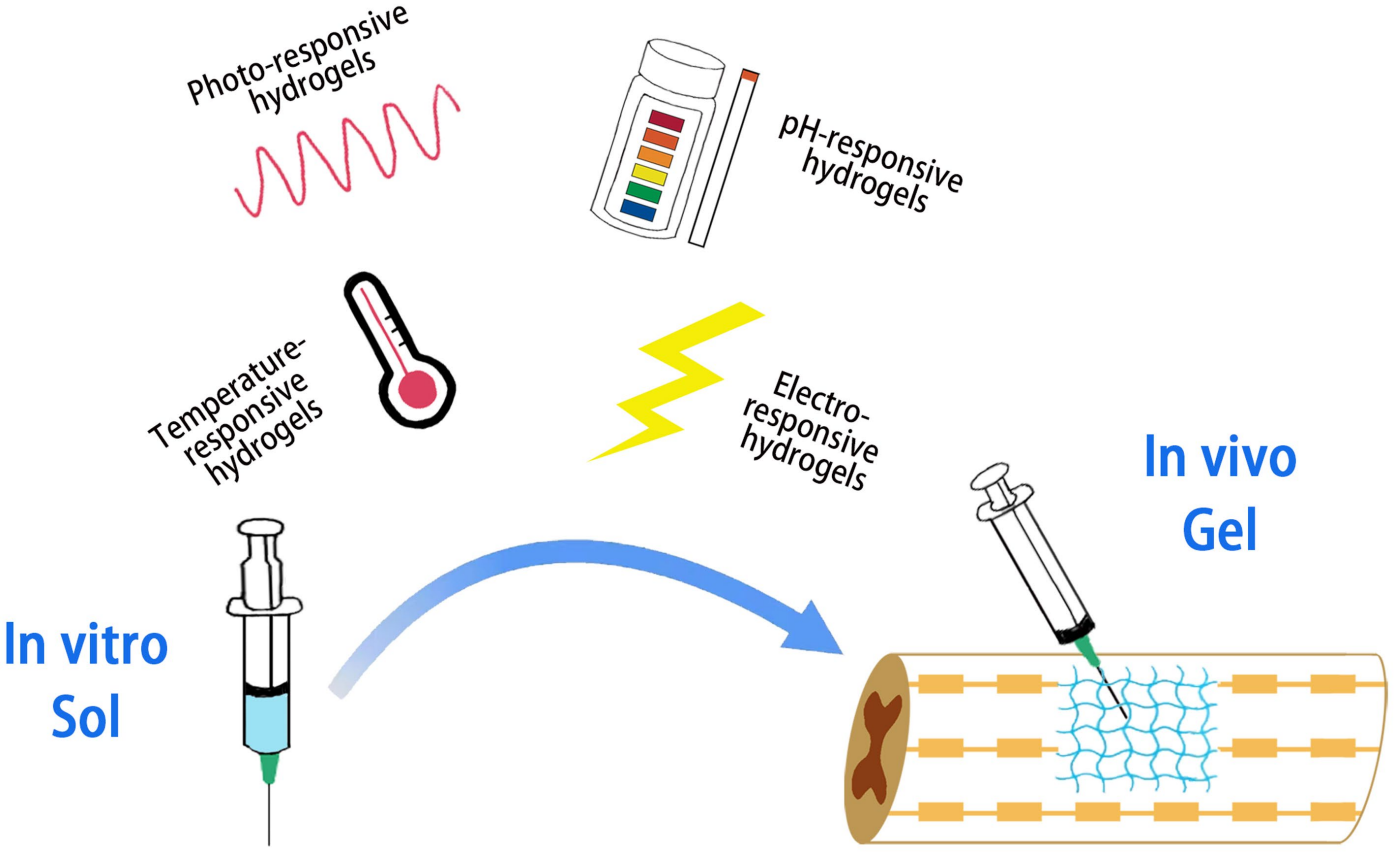
Smart hydrogels responding to different stimuli. The hydrogel is capable of performing a sol–gel or gel–sol transition in response to external stimuli.

Hydrogel molecules are crosslinked either physically or chemically. Chemical crosslinking is formed through covalent bonds between molecules, and the crosslinking state is stable and difficult to change. Physical cross-linking pathways are non-covalent bonds promoted by ionic interactions, hydrogen binding or hydrophobic interactions, and the cross-linking state changes with external conditions (e.g., light, temperature, pH, etc.), which is dynamically reversible (Silva et al., 2021). Supramolecular hydrogels are a special class of physically cross-linked hydrogels that are able to respond to external stimuli and undergo sol–gel/or gel–sol transition in response to subtle changes in the environment and are known as smart hydrogels (Figure 3). Such hydrogels have a wide range of applications in tissue engineering, cell and drug delivery, and regulation of the tissue environment to promote innate tissue repair (Hoque et al., 2019; Psarrou et al., 2023).

#### 3.2.1. Temperature-responsive hydrogels

Temperature-responsive hydrogels undergo sol–gel or gel–sol transitions with subtle changes in ambient temperature, and their temperature-dependent phase transitions are mainly driven by hydrophilic and hydrophobic interactions. Near the critical temperature, hydrophilic polymers lose a large amount of water and form gels dominated by hydrophobic interactions (Rahmani et al., 2023). ZHENG et al. prepared poly (lactic acid)-poly (ethylene glycol)-poly (lactic acid) (PLA–PEG–PLA) as injectable temperature-sensitive hydrogel materials, which successfully inhibited the expression of inflammatory cytokines in the early stage of injury, reduced the apoptosis of neurons in spinal cord injured rats and promoted the functional recovery (Zheng et al., 2021).

#### 3.2.2. Light-responsive hydrogels

Photoresponsive hydrogels utilize hydrogel factors encoded by photosensitive groups (e.g., photocracking groups and photoisomerization groups) to trigger phase transitions (Hoque et al., 2019). Wang et al. prepared F127 hydrogel loaded with extracellular vesicles for SCI treatment. F127 is a copolymer of poly(citrate-polyethyleneimine), which is activated in the presence of rapidly cross-linked and cured into gels by UV and visible light in the presence of a photoinitiator. This delivery system has been shown to inhibit fibrotic scar formation, reduce inflammation, and promote myelin and axonal regeneration (Wang et al., 2021).

#### 3.2.3. pH-responsive hydrogels

For hydrogels used in SCI therapy, in addition to good loading capacity and long duration retention *in vivo*, the ability for time-specific controlled release is required. pH-responsive hydrogels have many acidic or basic groups that can rapidly receive or release protons in the environment, thus enabling an intelligent response to solution pH. It is stable under neutral conditions but can be degraded in the inflammatory microenvironment of SCI with low pH to achieve pH-responsive drug release from the inflammatory site of SCI (Chang et al., 2022). A pH and photothermal dual-responsive drug-loaded nanoparticle, injectable collagen hydrogel system was fabricated to treat SCI. The system uses ZIF-8 as the carrier material, a ligand compound formed by the self-assembly of the ligand 2-methylimidazole and a zinc-based compound, which has good drug release in acidic inflammatory sites and maintains a stable state until it reaches the affected area. Hydrogel degradation was able to raise the pH of the microenvironment，promote proliferation of neural stem cells and protect neurons. The loaded drug paclitaxel played an effective role in promoting axonal lengthening and neuronal differentiation. The results showed that the system resulted in higher BBB scores in rats with SCI (Chen and Dai, 2023). Rogina et al. prepared a pH-responsive chitosan-HAP hydrogel with NaHCO_3_ as the gelling agent, which not only achieved rapid gelation of chitosan-HAP-based hydrogels within 4 min, but also served a pH-passive targeting function to react with the micro-acidic environment and achieve drug release (Rogina et al., 2017).

#### 3.2.4. Electrically responsive hydrogels

Electrically charged drug molecules under an electric field move in the opposite direction of charge, which is the key mechanism of drug release from electrically responsive hydrogels. These gels constructed from conductive polymers such as polypyrrole and polyaniline also contract under electrical stimulation to induce drug release (Mo et al., 2022). Conductive hydrogels have promising applications in the repair of electrically active biological tissues such as cardiac muscle, skeletal muscle and nerves. This is because conductive hydrogel matrices can improve electrical signaling between adjacent cells, thus facilitating the reconstruction of tissue motor and sensory signals (Kiyotake et al., 2022; Feng et al., 2023). Proper electrical conductivity facilitates neurite growth, and better axon regeneration length, axon growth rate, and axon remyelination formation were observed at conductivity of 1–10 s/m (Zhou et al., 2018). Cai et al. developed a unique and simple agarose/gelatin/polypyrrole (Aga/Gel/PPy, AGP3) hydrogel with electrical conductivity and modulus similar to that of spinal cord similarly. Gelation occurs through non-covalent interactions and physical cross-linking characteristics make AGP3 hydrogel injectable. *In vitro* cultures showed that AGP3 hydrogel exhibited excellent biocompatibility and promoted the differentiation of NSCs toward neurons while inhibiting the over proliferation of astrocytes. *In vivo* implantation of AGP3 hydrogel completely covered the tissue defects and reduced the injured luminal area (Yang B. et al., 2022; Yang S. et al., 2022).

### 3.3. Application of hydrogel scaffolds for SCI repair

**Figure 4:**
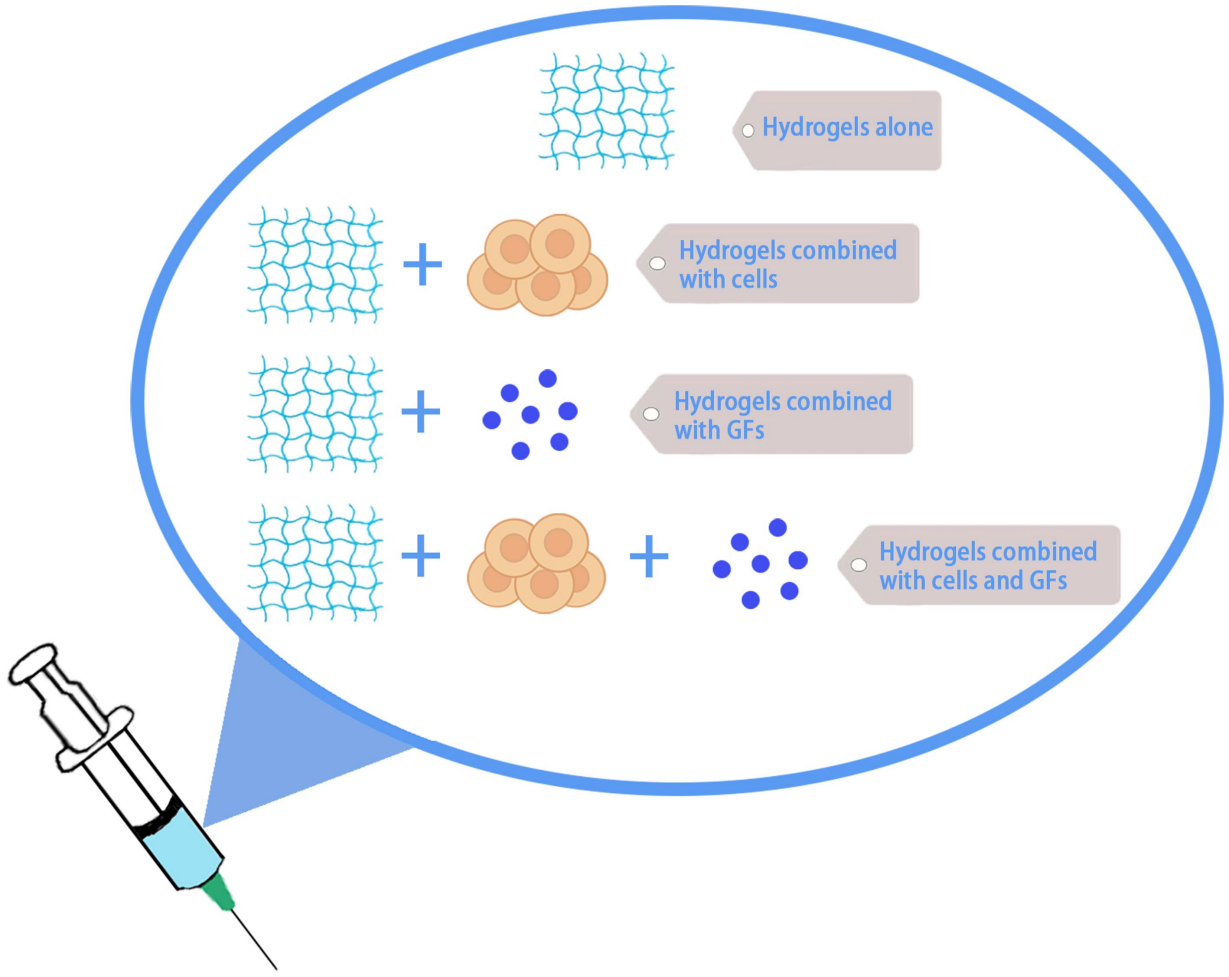
Hydrogel therapy for SCI. Hydrogel can be used alone or in combination with cells, growth factors, etc., for the treatment of SCI.

So far, a lot of research on hydrogel application on central nerve repair has been carried out. For example, the construction of extracellular matrix (ECM) by hydrogel bridging the gap between lesion sites can promote axon growth into the hydrogel matrix and enhance motor neuron innervation in the lumbar spinal cord (Liu T. et al., 2022; Park et al., 2022). Zhang et al. developed an injectable hydrogel with mechanical properties similar to those of spinal cord and sustained prolonged drug delivery, which was able to reduce apoptosis and promote nerve regeneration, promoting significant recovery of motor and voiding functions in rats with SCI (Zhang et al., 2022). However, due to the complex pathological mechanism of SCI, hydrogel treatment alone does not restore neurological function completely, and combined biologic factor and cellular hydrogel treatment is more effective than single treatment. Current hydrogel-based tissue engineering strategies involving scaffolds, cells, small molecules, and growth factors hold promise for SCI treatment (Li et al., 2022; Khaing et al., 2023) (Figure 4).

#### 3.3.1. Combined action of hydrogels and neurotrophic factors

Neurotrophic factors mainly include nerve growth factor (NGF), brain-derived neurotrophic factor (BDNF), neurotrophic factor-3 (NT-3), and neurotrophic factor-4 (NT-4). In recent years, hydrogels combined with neurotrophic factors have been increasingly used in SCI repair, and studies have shown that neurotrophic factors can improve neuronal survival in the area of SCI, induce differentiation of stem cells to neuronal cells, promote axonal regeneration, have anti-apoptotic, and mediate cell differentiation (Park et al., 2022). For example, temperature-sensitive hydrogels loaded with NGF can enhance neuroprotection and promote neuroregeneration in rat spinal cord contusion models by inhibiting apoptosis in response to endoplasmic reticulum stress (Zhao et al., 2016). This composite tissue engineering scaffold can not only bridge the lesion site, but also promote the endogenous regeneration process through various signaling pathways (Madhusudanan et al., 2020). He et al. designed a novel anti-inflammatory peptide and BDNF-modified hyaluronic acid-methyl-fiber hydrogel to promote neural regeneration in rats with SCI, which had a small swelling rate and sustained release of BDNF. Compared with hydrogel treatment alone, the hydrogel combined with BDNF significantly improved neurological function and histomorphology, reduced inflammatory cytokine expression and cavitation, and increased neuronal survival in rats with SCI (He et al., 2019; Huang et al., 2021). NT-3 promoted the recovery of motor neurons after SCI and the sprouting of downstream native spinal cord neurons, protected the downstream spinal cord tract, and improved hindlimb motor function. Li et al. applied natural biomaterial-chitosan complex neurotrophic factors (NTs) to a series of novel active biomaterials, which could release neurotrophic factors in a controlled manner for a long period of 14 weeks, improve the local microenvironment of the injury area, activate endogenous neural stem cells and recruit them to migrate to the injury site, guiding them to differentiate into mature neurons. The newborn neurons could form functional neural loops with host cells eventually allowing functional recovery (Hao et al., 2017; Wang et al., 2023). In addition, encapsulation of NT-3 in PLGA nanoparticles combined with methylcellulose and hyaluronic acid injectable hydrogel for SCI can achieve slow release of NT-3 into the environment where dorsal root ganglion cells survive and achieve long-term sustained stimulation of nerve cells (Stanwick et al., 2012). It is worth noting that the release of NT-3 is closely related to the degradation time of hydrogels, and the development of hydrogels with appropriate degradation rate combined with neurotrophic factors can be more beneficial for SCI repair.

#### 3.3.2. Combined action of hydrogels and stem cells

Cell transplantation alone has inevitable problems such as immune rejection and the inability of cell flow to remain in the damaged area for a long period of time, making it impossible to treat the injured area in a sustainable manner. By combining hydrogels and cells, it is possible to stabilize the cells at the site of injury while enhancing the effect of hydrogels and better simulating the ECM environment (Suzuki et al., 2023). Compared with adult cells, stem cells have more potential applications, and cells commonly used in central nervous system injury repair cell therapy include NSCs/neural group cells (NPCs), induced pluripotent stem cells (iPSCs), and MSCs (He et al., 2022; Silva et al., 2023).

NSCs/NPCs can differentiate into astrocytes, oligodendrocytes, and neurons to build new neural networks, increase neuronal connectivity and achieve functional recovery. Transplantation of stromal glia loaded with NSCs such as the SCI area in rats was able to detect new neuronal regeneration while promoting improved motor function in rats (Wang et al., 2020). Compared with the simple transplantation of NSCs, the injection of 3D printed biomimetic hydrogel combined with NSCs into the SCI rat model can make the NSCs have a higher survival rate, and can well mimic the natural structure in the spinal cord and promote axonal regeneration (Ashammakhi et al., 2019; Koffler et al., 2019). In further studies, it was found that developing hydrogel-loaded NSCs that mimic the natural microenvironment has better therapeutic effects, and ECM proteins may help regulate integrin expression and regulation of AKT/ERK-related signaling pathways in NSCs, promoting proliferation and differentiation of NSCs and formation of new neural networks in SCI-injured regions (Xu et al., 2021).

IPSCs are pluripotent stem cells that can differentiate into therapeutic target cells such as neurons and glial cells when stimulated by different cellular microenvironments. IPSCs are often used as an alternative to NSCs in SCI tissue engineering repair, mainly because iPSCs can differentiate into NSCs by induction and can be cultured in large numbers *in vitro* without ethical implications (Ramotowski et al., 2019). A study by Kong et al. generated iPSC from dermal fibroblasts and transplanted their derived NSCs into SCI rat lesions, which effectively suppressed inflammation and reduced fibrotic tissue (Kong et al., 2021). Overall, iPSCs are already capable of being derived into NSCs and are similar to NSCs in terms of therapeutic effects, but their potential for multidirectional differentiation, especially to tumorigenicity, limits their application in tissue engineering hydrogels (Nagoshi et al., 2020).

MSCs are a kind of dominant seed cells with multi-directional differentiation potential, immune regulation, and biological anti-inflammatory effects, which can differentiate into many cell types such as bone, cartilage, neurons, and myocardium. They also have the advantages of easy access and preservation. Currently, they are widely used in SCI tissue engineering repair. By combined with hydrogels, MSCs are able to release vesicles with anti-inflammatory and antioxidant properties that can enhance the survival and proliferation of endogenous NSCs, thus promoting recovery from SCI (Boido et al., 2019; Zhang et al., 2020). MSCs can differentiate into neurons, but unlike NSCs, they have immature characteristics, and hydrogel combined with MSCs is more valued for the anti-inflammatory and pro-angiogenic properties of MSCs.

#### 3.3.3. Combined action of hydrogels and small molecules

In the organism, cells encounter a large number of soluble small molecules from the aqueous microenvironment. Small molecules are distributed in the microenvironment on which the cells depend and are produced by cellular autocrine or paracrine secretion, with a fraction of small molecules produced by endocrine secretion in the circulatory system. These small molecules either diffuse freely in aqueous media or are fixed in the ECM and are tightly controlled in space and time. The local concentration, spatial distribution and biological activity of small molecules play an important role in the regulation of different cellular behaviors. VEGF has been shown to promote the proliferation of neuronal precursors, and injection of hydrogels loaded with VEGF into SCI sites significantly promoted angiogenesis and axonal growth, contributing to the restorative effects of the hydrogels (des Rieux et al., 2014). Furthermore, the addition of Transforming Growth Factor-β1 to hydrogels stimulates fibroblast differentiation to myofibroblasts, which contributes to wound healing and fibrosis development (Park et al., 2022). Some small molecule drugs are also frequently loaded on hydrogels for SCI therapeutic studies, such as serine protease (Kwiecien et al., 2020), cabazitaxel (Li X. L. et al., 2019), minocycline and paclitaxel (Nazemi et al., 2020).

Compared to single growth factors, exosomes seem to be more popular. Exosomes are extracellular vesicles secreted by cells, which are rich in various proteins and miRNAs and are widely involved in regulating various cellular pathways (Liu et al., 2021). In addition to anti-inflammation and pro-angiogenesis, exosomes can also activate neural stem cells and induce differentiation into neurons. Li et al. (2021) found that hippocampal niche exosomes can promote the differentiation of neural stem cells into neurons, and further studies showed that miR-3,559-3P and miR-6,324 in hippocampal niche exosomes have a pro-NSC differentiation effect (Cheng et al., 2021). Not only as small molecules used in tissue engineering for SCI treatment, exosomes can also be used as carriers to load siRNA, miRNA and antagonists and deliver them to the site of injury through the “homing” function of exosomes, so that the hydrogel can play a better therapeutic role (Huang et al., 2020).

Compared with MSCs, MSCs exosomes (MSCs-Exo) not only have anti-inflammatory and homing properties, but also can pass through the blood-spinal cord barrier to exert a therapeutic effect. Hydrogels combined with MSCs-Exo also reduced inflammation, enhanced neural stem cell recruitment, and promoted axon regeneration (Li et al., 2020), while overcoming some disadvantages of hydrogels, such as the aggravating effect of conductive hydrogels on inflammation at the injured site (Fan et al., 2022). It has more advantages than MSCs in the treatment of SCI (Table 2).

**Table 2:** Application of hydrogel scaffolds for SCI repair.

Application forms	Cells or drugs	Functions	Drawbacks
Hydrogel + NTs	NGF (Zhao et al., 2016)	Inhibit apoptosis, enhance neuroprotective effects and promote neuroregeneration	
Hydrogel + NTs	BDNF (Huang et al., 2021)	Improve nerve function, reduce inflammatory cytokines and cystic cavitation	
Hydrogel + NTs	NT-3 (Wang et al., 2023)	Promote the sprouting of spinal cord neurons and protect the downstream spinal cord tract	
Hydrogel + stem cells	NSCs/NPCs (Xu et al., 2021)	Regulate integrin and AKT/ERK signaling pathways and promote new neural network formation	
Hydrogel + stem cells	iPSCs (Kong et al., 2021)	Inhibit inflammation and reduce fibrotic tissue	Potential tumorigenicity
Hydrogel + stem cells	MSCs (Zhang et al., 2020)	Release exosomes, promote the survival and proliferation of endogenous NSCs and pro-angiogenesis	MSCs differentiate into neurons, but immature
Hydrogel + small molecules	VEGF (Des Rieux et al., 2014)	Promote proliferation of neuronal precursors, angiogenesis and axonal growth	
Hydrogel + small molecules	TGF-β1 (Park et al., 2022)	Stimulate differentiation of fibroblasts into myofibroblasts and promote wound healing	
Hydrogel + small molecules	Exosomes (Liu et al., 2021)	Widely involved in regulating various cellular pathways, anti-inflammatory, pro-angiogenic and good delivery properties	

### 3.4. Relevant clinical trials

At present, the clinical treatment of SCI is still mainly based on traditional drugs and surgery, but some clinical trials using new treatment methods have been carried out (Table 3).

**Table 3:** Relevant clinical trials and results.

Implants	Therapeutic model	Results
MSCs+SCs (Oraee-Yazdani et al., 2021)	Cell transplantation alone	Statistical improvement in patient sensory and neurological function; safety assessment showed no systemic complications
G-CSF (Koda et al., 2021)	Biological factor treatment alone	Early clinical trials showed safety and potential efficacy; a secondary endpoint in phase 3 clinical trials showed higher AIS scores in the G-CSF group
PLGA-PLL (Kim et al., 2022)	Hydrogel treatment alone	Significant improvement in 44% of patients within 6 months, with most patients improving over a longer time frame; no neurological adverse effects
MSCs+CS (Deng et al., 2020)	Cell-hydrogel combined treatment	AIS and ADL scores elevated, bowel and urinary function restored; new nerve fiber connections formed; no serious complications observed
BMMCs+CS (Chen et al., 2020)	Cell-hydrogel combined treatment	Improvement in superficial sensory and autonomic function; scaffold implantation supports continuity of damaged spinal cord; no associated adverse effects observed
BM-MSCs+GM-CSF (Park et al., 2005)	Cell-biological factor combined treatment	Postoperative improvement in both sensation and movement; combination therapy better than GM-CSF alone; GM-CSF side effects observed
BMCs+GM-CSF (Yoon et al., 2007)	Cell-biological factor combined treatment	Increased AIS grading in acutely patients with no significant improvement in the chronically group; increased neuropathic pain during treatment remains to be studied

MSCs and Schwann cells (SCs) have safety as well as complementary properties and are considered to be two of the best candidates for SCI treatment. A clinical trial of intrathecal combination of autologous bone marrow MSCs and SCs in 11 SCI patients revealed statistically significant improvements in sensory and neurological function. Safety assessments showed no systemic complications. It appeared that the use of this cell combination was safe and effective for clinical applications in subacute phase spinal cord regeneration (Oraee-Yazdani et al., 2021).

Early clinical trials have demonstrated the safety, feasibility and suggestive efficacy of granulocyte colony-stimulating factor (G-CSF) in the treatment of SCI. To confirm this efficacy and to approve G-CSF for the treatment of SCI, a phase 3 clinical trial was conducted, designed as a prospective, randomized, double-blind and placebo-controlled comparative trial. The trial included 88 patients. A secondary endpoint showed that the American Spiral Injury Association Impairment Scale (AIS) scores tended to be higher in the G-CSF group at 6 months (*p* = 0.062) and 1 year (*p* = 0.073) after administration compared to the placebo control group (Koda et al., 2021).

A clinical trial implanted spinal nerve scaffolds, which comprises poly (lactic-co-glycolic acid)-b-poly-(L-lysine) (PLGA-PLL), in 16 patients with SCI. 44% of the patients showed significant improvement within 6 months, and most patients had an additional AIS score increase at 12 months and beyond. Patients remained stable and no long-term neurological problems were noted at 24-month follow-up. These data further support the safety and possible benefit of hydrogel scaffold implantation in this patient population (Kim et al., 2022).

Forty patients with acute SCI were recruited and randomly divided into the treatment group and the control group. The treatment group (*n* = 20) received a collagen scaffold (CS) loaded with MSCs from neonatal umbilical cord tissue; the control group (*n* = 20) did not receive the stem cell-loaded collagen implant. All patients were followed up for 12 months. In the treatment group, AIS scores and activities of daily living scores were elevated, bowel and urinary function were restored, and residual urine output was reduced. In addition, magnetic resonance imaging showed the formation of new nerve fiber connections and diffusion tensor imaging showed recovery of electrophysiological activity after treatment. No serious complications were observed during follow-up. In contrast, neurological function in the control patients did not improve during the follow-up period. The above data provide preliminary evidence that transplantation of human umbilical cord MSCs onto collagen scaffolds can promote recovery of neurological function after acute SCI (Deng et al., 2020).

Chen et al. implanted collagen scaffolds containing autologous bone marrow mononuclear cells (BMMC) into 7 patients with SCI at clean sites with up to 3 years of follow-up. During follow-up, no adverse symptoms associated with stem cell or functional scaffold implantation were observed. In addition, partial improvement in superficial sensory and autonomic function was observed in some patients. Magnetic resonance imaging demonstrated that the scaffold implantation supported the continuity of the damaged spinal cord after treatment. These findings suggest that implantation of a collagen scaffold combined with stem cells may be a safe and promising clinical treatment for patients with acute complete SCI (Chen et al., 2020).

Park et al. evaluated the therapeutic efficacy of autologous bone marrow mesenchymal stem cells (BM-MSCs) transplantation combined with granulocyte-macrophage colony-stimulating factor (GM-CSF) in 6 patients with complete SCI. BM-MSCs transplantation at the site of injury and subcutaneous GM-CSF administration were performed in five patients. One patient was treated with GM-CSF only. The follow-up period was 6 to 18 months. Sensory and motor improvements were noted postoperatively. Five patients had elevated AIS scores, showing improved neurological function. The last patient maintained an AIS grade of A. No immediate worsening of neurological symptoms was noted. Side effects of GM-CSF treatment, such as fever and myalgia, were noted. No serious complications that increased mortality and morbidity were noted (Park et al., 2005).

To evaluate the safety and therapeutic efficacy of autologous human bone marrow cell (BMC) transplantation and GM-CSF, a phase I / II open-label and non-randomized study was conducted in 35 patients with complete SCI. 30.4% of acutely and subacutely treated patients had increased AIS grading, while no significant improvement was observed in the chronically treated group. Increased neuropathic pain during treatment remains to be studied. Long-term and large-scale multicenter clinical studies are needed to determine its precise therapeutic effects (Yoon et al., 2007).

As mentioned above, cell therapy, factor therapy or hydrogel therapy alone each has been shown effective in clinical trials for SCI. However, due to changes in the microenvironment at the injury and the explosive release and rapid degradation of fluids, a combination of biomaterial delivery systems is needed for better efficacy (Lv et al., 2021; Yuan et al., 2021b). Combination therapy with cells and hydrogels has demonstrated the superiority of hydrogel delivery systems, which have a good performance in clinical treatment. In addition, the combination of cells and factors has been shown to be more efficacious than treatment alone, but some adverse effects remain, such as fever, myalgia and neuropathic pain seen in the clinical trials described above, which may be related to the rapid release or off-target effects of cells and factors. Transplantation with hydrogel-loaded cells and factors may address these issues, but no such attempts have been made in clinical trials for the time being.

### 3.5. Novel strategies for SCI

With the continuous development of technology, hydrogels are not only used as fillers but also as carriers of drugs/factors and cells to promote tissue growth (Walsh et al., 2022; Pourkhodadad et al., 2023). Combining hydrogels with cytokines and seed cells to equip scaffolds with more functions is a new tissue engineering strategy for SCI treatment. Liu et al. developed a 3D-printed hydrogel that loaded NSCs and OSMI-4, an O-GlcNAc transferase inhibitor, and transplanted it into the injured site of SCI rats. This significantly promoted neuron regeneration, axon growth and motor function recovery in rats, showing a better therapeutic effect than the ordinary combination therapy (Liu X. et al., 2022). This combination of hydrogels, cells, and active factors to promote spinal cord regeneration is currently considered the most effective way to treat SCI, and more tissue engineering scaffolds based on this strategy will be available for SCI treatment in the future.

## 4. Discussion

SCI is a medical problem that affects millions of people around the world and needs to be solved urgently, but traditional surgery and medication therapies are difficult to fundamentally cure it. The microenvironmental homeostasis at the injury site is imbalanced and unsuitable for cell growth. Traditional systemic drug delivery is limited by the blood-spinal cord barrier, and the therapeutic effect is unsatisfactory. In this paper, we introduce a highly biocompatible material hydrogel with 3D mesh structure, which can effectively guide the reconstruction of axons and also act as a carrier for drugs or cells, providing a good environment and controlling their release. Smart hydrogels can respond to different stimuli such as temperature, light and pH to complete the sol–gel transition, and thus can be used for *in situ* injection. The single-component hydrogels are still lacking in some aspects such as cell adhesion, modifiability and slow-release ability, and the composite hydrogels with the addition of nanoparticles, nanotubes, slow-release microspheres, etc. can make up for these defects. Current advances in stem cell and neuroregenerative science research hold the promise of a complete cure for SCI. A meta-analysis based on 62 clinical trials found that although the efficacy of cell therapy is encouraging, its adverse effects are still a concern (Shang et al., 2022). Hydrogels have been shown in clinical trials to be used in combination with stem cells in the treatment of SCI to improve the microenvironment at the injury, provide support for cell proliferation and migration, and compensate for the inadequacy of cell transplantation therapy alone. Numerous studies to date have shown significant efficacy of combined treatment with cells, hydrogels, and active factors in animal SCI models (Feng et al., 2022; Saremi et al., 2022; Li et al., 2023). However, few clinical trials have been conducted to treat SCI patients with a combination of these three. The therapeutic approach of hydrogel-loaded cells and active factors still has a long way to go from animal experiments to clinical application. Firstly, the quality of the composite scaffolds needs to be ensured. The production of hydrogel scaffolds for human transplantation requires stricter manufacturing practices and quality control. Secondly, in order to achieve better efficacy, the combination of hydrogels, cells, and factors in terms of types and quantities needs to be explored. For composite hydrogels, the adjustment also includes many aspects like materials and physical and chemical properties. Another point of concern is that the current clinical trials have not shown serious adverse reactions like tumor formation, but may be due to the lack of follow-up time. Therefore, continuous follow-up is needed to strictly control the invasion while hydrogel scaffolds guide cell migration.

## Author contributions

MC, LC, and TW contributed substantially to the article concept and manuscript writing. YL, JZ, and XZ retrieved the literature and reviewed the manuscript. ZL and HW revised and approved the final version before submission. All authors have participated actively in the study and have read and approved the final manuscript.

## Funding

This research was funded by the National Natural Science Foundation of China (No. 82071374), Discipline construction project of Guangdong Medical University (No. 1.13), Dongguan Science and Technology of Social Development Program (No. 20221800905682), Talent Development Foundation of the First Dongguan Affiliated Hospital of Guangdong Medical University (Nos. PF100-1-01 and PF100-1-04), College Students Innovative Experimental Project in Guangdong Medical University (Nos. FYDB015, ZCDS001, ZYDB016, ZZDI001, FZDB005, ZZDC001, and ZYDS002) and College Students’ Science and Technology Innovation Training Project (Nos. 202210571025, 202210571030, S202210571087, and S202210571097).

## Conflict of interest

The authors declare that the research was conducted in the absence of any commercial or financial relationships that could be construed as a potential conflict of interest.

## Publisher’s note

All claims expressed in this article are solely those of the authors and do not necessarily represent those of their affiliated organizations, or those of the publisher, the editors and the reviewers. Any product that may be evaluated in this article, or claim that may be made by its manufacturer, is not guaranteed or endorsed by the publisher.
